# Dynamic evolution of emphysema and airway remodeling in two mouse models of COPD

**DOI:** 10.1186/s12890-021-01456-z

**Published:** 2021-04-26

**Authors:** Yue Yang, Tingting Di, Zixiao Zhang, Jiaxin Liu, Congli Fu, Yan Wu, Tao Bian

**Affiliations:** 1 Department of Respiratory Medicine, Wuxi People’s Hospital Affiliated With Nanjing Medical University, Wuxi, 214023 Jiangsu People’s Republic of China; 2 Respiratory and Critical Care Medicine, Zhejiang Province People’s Hospital, Hangzhou, 310000 Zhejiang People’s Republic of China

**Keywords:** Emphysema, Airway remodeling, Chronic obstructive pulmonary disease, Cigarette smoke, Lipopolysaccharide

## Abstract

**Background:**

Establishment of a mouse model is important for investigating the mechanism of chronic obstructive pulmonary disease (COPD). In this study, we observed and compared the evolution of the pathology in two mouse models of COPD induced by cigarette smoke (CS) exposure alone or in combination with lipopolysaccharide (LPS).

**Methods:**

One hundred eight wild-type C57BL/6 mice were equally divided into three groups: the (1) control group, (2) CS-exposed group (CS group), and (3) CS + LPS-exposed group (CS + LPS group). The body weight of the mice was recorded, and noninvasive lung function tests were performed monthly. Inflammation was evaluated by counting the number of inflammatory cells in bronchoalveolar lavage fluid and measuring the expression of the IL-6 mRNA in mouse lung tissue. Changes in pathology were assessed by performing hematoxylin and eosin and Masson staining of lung tissue sections.

**Results:**

The two treatments induced emphysema and airway remodeling and decreased lung function. Emphysema was induced after 1 month of exposure to CS or CS + LPS, while airway remodeling was induced after 2 months of exposure to CS + LPS and 3 months of exposure to CS. Moreover, the mice in the CS + LPS group exhibited more severe inflammation and airway remodeling than the mice in the CS group, but the two treatments induced similar levels of emphysema.

**Conclusion:**

Compared with the single CS exposure method, the CS + LPS exposure method is a more suitable model of COPD in airway remodeling research. Conversely, the CS exposure method is a more suitable model of COPD for emphysema research due to its simple operation.

**Supplementary Information:**

The online version contains supplementary material available at 10.1186/s12890-021-01456-z.

## Introduction

Chronic obstructive pulmonary disease (COPD) is a chronic progressive lung disease characterized by a persistently limited airflow, chronic airway inflammation, airway remodeling and emphysema [1]. It is the fourth leading cause of morbidity and mortality worldwide [2]. Exacerbations and comorbidities of COPD contribute to a substantial burden on patients and society [3]. Currently, efficient pharmacological therapies are unavailable for COPD, and thus new therapeutic drugs that will improve patient outcomes are urgently needed [4]. An appropriate animal model is important and indispensable for further study of this disease.

Mice are always suitable animals to establish COPD models due to their genetic similarity to humans, ease of breeding, high survival rate and extensive study [5, 6]. Because exposure to cigarette smoke (CS) is an independent risk factor for COPD [7, 8], long-term CS exposure is one of the widely accepted methods for modeling COPD [9–11]. Whole-body CS exposure and whole-body CS exposure combined with an intranasal instillation of lipopolysaccharide (LPS) are the two most frequently applied methods, because they are relatively consistent with the clinical condition and the two models have been compared. However, based on previous studies [11–13], each pathological feature might appear at different time points after model establishment, and the severity of pathological changes might increase over time. In the present study, we first evaluated and compared the dynamic evolution of the pathology and lung function in these two most widely used mouse models of COPD to provide evidence for further COPD modelling. Female mice have been shown to be more susceptible to COPD; however, a greater proportion of men than women are smokers, and thus we chose male mice as our experimental subjects.

## Materials and methods

### Animal models

Wild-type C57BL/6J mice (6–8 weeks old) were purchased from Changzhou Cavans Animal Experiment Co., Ltd. (Changzhou, China). Animals were housed in a temperature- (22 ± 2 °C) and humidity-controlled room with free access to both fresh water and standard laboratory food in WuXi People’s Hospital Animal Experiment Center. After 1 week of conditioning on a 12 h light:dark cycle, 108 mice were randomly divided into the following three groups: the (1) control group, (2) CS group (CS exposure in a homemade tempered glass box), and (3) CS + LPS group (CS exposure combined with an intranasal instillation of LPS (7.5 μg/50 μl, Sigma L2880, USA) on the 1st and 14th days). The weight of each mouse was recorded using the same electronic scale every Saturday between 6 and 7 p.m., and the weight gain was analyzed from the monthly weight data.

The animals used in this study were maintained in accordance with the ethical guidelines of the International Council for Laboratory Animal Science (ICLAS) and the Policy of Animal Care and Use Committee of Nanjing Medical University. Humane care was provided according to the 3R principles of animal experiments.

### Apparatus

**Fig. 1:**
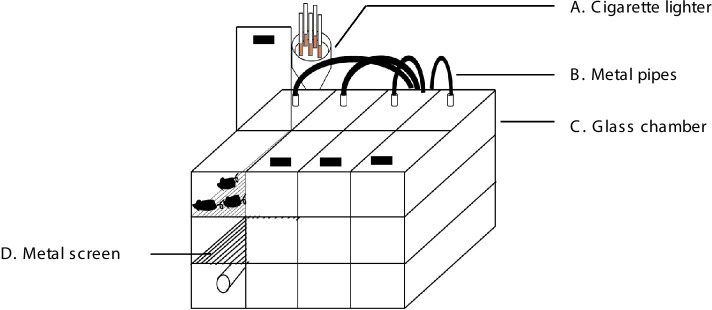
The custom-made apparatus for the establishment of COPD in mice

The CS exposure apparatus consisted of a cigarette lighter, turbo fans, a glass chamber and metal pipes. The metal pipes connected the other three parts, transporting smoke from the cigarette lighter (with 20 holes for cigarettes to be inserted) to the glass chamber and then outside. Smoke was pumped from a cigarette lighter to the tank though two filter screens, the density of which as adjusted by adjusting the voltage of the turbo fan. The size of the glass chamber was 100 cm (length) × 65 cm (width) × 50 cm (height) and it was separated into three layers in height and four equal smaller chambers along its length. No more than 30 mice were placed in the top layer of each chamber (the smoke in each chamber had the same concentration) (Fig. 1).

### Modelling procedures

**Fig. 2:**
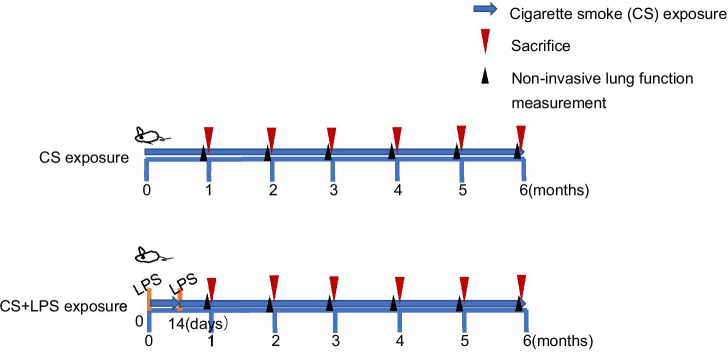
The procedures used to model COPD

Commercial filtered cigarettes (DAQIANMEN, from the Shanghai Tobacco Industrial Co., Ltd., China) containing 10 mg of tar and 0.8 mg of nicotine per cigarette were used in this study. After the 1 week adaptation period, mice in the CS and CS + LPS groups were placed in the chamber and exposed to CS (10 cigarettes per chamber for 1 h, 2 h each session, twice per day, 7 days per week). The mice in the control group were placed in the same environment when mice in the CS and CS + LPS groups were exposed to CS. The details of modelling procedures are provided below. In step 1, mice in the CS group and CS + LPS group were randomly placed in 2 glass chambers (the other 2 chambers not in use were sealed). Tape was used to close the gap. In step 2, 20 cigarettes were inserted into the cigarette lighter, the smoke intake turbo fan was switched on at 10 V and the exhaust turbo fan was switched on at 3 V. Then, 5 cigarettes were lit to fill the chambers with smoke. In step 3, when the first 5 cigarettes had extinguished, the voltage of the smoke intake turbo fan was set to 5 V and the exhaust turbo fan was switched off before another 3 cigarettes were lit, with the middle hole in the cigarette lighter remaining empty. In step 4, when the 3 cigarettes were extinguished, the voltage of the smoke intake turbo fan was set to 10 V again, and then another 3 cigarettes were lit, with the middle hole in the cigarette lighter sealed. Steps 3 and step 4 were repeated until all 20 cigarettes had burned. In step 5, the voltage of the smoke intake turbo fan was set to 5 V and the middle hole remained empty until most of the smoke in the chamber had cleared. All of the CS was pumped out, and fresh air was pumped in after 1 h of exposure. Mice in the CS + LPS group were exposed to smoke using the same procedure as the CS group, except for on days 1 and 14. Mice in the CS + LPS group were administered an intranasal instillation of LPS (7.5 µg/50 µl, Sigma L2880) on days 1 and 14 [14]. Six randomly selected mice in each group were sacrificed at 1, 2, 3, 4, 5, and 6 months after model establishment (Fig. 2).

### Lung function tests

Mouse lung function was measured monthly in the Jiangsu Provincial Center for Disease Control and Prevention using whole-body plethysmography (Buxco Electronics, Ltd., USA) according to the manufacturer’s protocol. Briefly, mice were placed randomly in a chamber connected to a sensitive pressure transducer, which measures slight pressure changes inside the chamber. The expiration time (Te), relaxation time (Tr), peak inspiratory flow (PIF), and peak expiratory flow (PEF) are all parameters reflecting restricted airflow. Enhanced pause (Penh, Penh = (Te/Tr − 1) × (PEF/PIF)) was recorded using FinePoint software (Buxco Electronics, Ltd., USA) when mice were quiet to evaluate pulmonary resistance. Values were averaged and reported as absolute Penh values.

### Collection of bronchoalveolar lavage fluid and cell counts

**Fig. 3:**
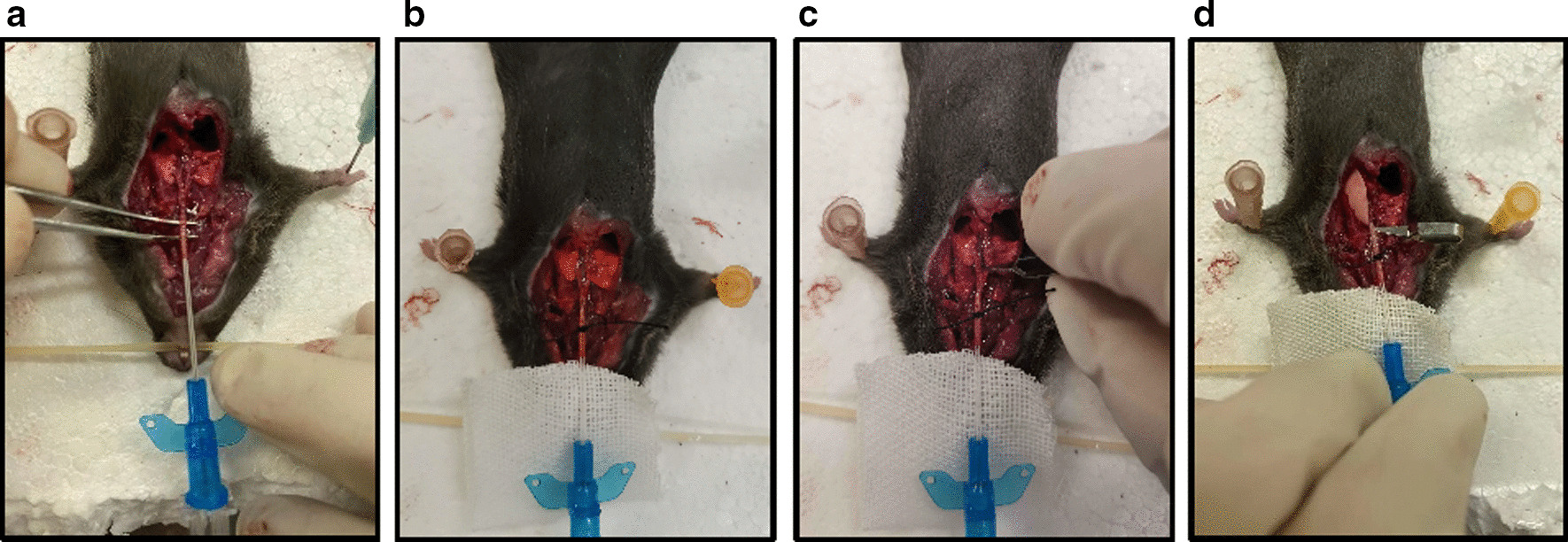
The procedures for sacrifice

As previously described [15], the mouse was anesthetized with intraperitoneal injection of 10% chloral hydrate. The mouse was put on a flat board, and its limbs and head were fixed to fully display the neck. Then, the skin and muscles of the anterior neck of each anesthetized mouse were incised to expose the trachea. The ribs were cut along the midline to expose the entire thorax, remove the heart, and separate the left and right main bronchial tubes. A 22G indwelling needle (0.9 × 25 mm) was inserted into the thyroid cartilage of the trachea at an angle of 30° (Fig. 3a) and fixed with 3.0 surgical suture (Fig. 3b). The right main bronchus was clamped with an arterial clip, and then a 1 ml syringe was attached to the indwelling needle (Fig. 3c). The left lung was slowly lavaged three times with 0.4 ml of ice-cold (Fig. 3d), sterile phosphate-buffered saline (PBS, Solarbio P1020, China). The recovery was more than 90% of the injected volume. The recovered solution was centrifuged at 2000 rpm for 10 min at 4 °C.

One hundred microliters of red blood cell lysis solution (Beyotime C3702, China) were used to resuspend the cell pellet and remove red blood cells, and the remaining cells in the bronchoalveolar lavage fluid (BALF) were collected and counted with a hemocytometer.

### Histological staining and morphological analysis

The left lobe and right lobes, except the right middle lobe, of the mouse lung were ligated, removed and quickly stored in liquid nitrogen for subsequent experiments. The right middle lobe of each mouse that was sacrificed in a specific month was slowly perfused with 0.2 ml of 4% paraformaldehyde and then fixed with 4% paraformaldehyde for 24 h. The fixed lung tissues were embedded in paraffin (Solarbio YA0014, China) and sectioned into 4 µm sections for further staining with hematoxylin and eosin (H&E) (Solarbio G1120, China) and Masson’s trichrome (Solarbio G1345, China). The slides were examined under a light microscope at a photograph documentation facility (Olympus BX53 8l48101, Tokyo, Japan) and the pictures were captured by CellSens Standard. The size of the alveolar space was examined by measuring the mean interalveolar septal wall distance (MLI) using Image-Pro Plus 6.0 software, as previously described [16]. Briefly, the MLI was measured by dividing the length of diagonals of 10 random fields of each lung tissue by the sum of intercepts counted within diagonals at 100 × magnification (the bronchial regions were not included). Collagen deposition around the bronchus was determined by the calculating the percentage of the collagen surface area (blue) in the whole image divided by the airway circumference using ImageJ software [12]. Collagen deposition around pulmonary arteries were determined by calculating the percentage of the collagen surface area (blue) around each artery. Vascular external diameter (ED) and vascular internal diameter (ID) were measured. The medial thickness (MT) = ED–ID, and the average medial thickness percentage (MT%) of the pulmonary arterioles was further calculated as MT% = (2 × MT–ED) × 100 [17].

### Western blotting

Mouse lung tissues were homogenized in ice-cold RIPA lysis buffer (CWBIO CW2333, China) with a complete protease inhibitor cocktail (CWBIO CW2200, China) and phosphatase inhibitor (CWBIO CW2383, China). Total protein was quantified by BCA protein assay (Beyotime P0010, China). Western blotting was performed as previously described [15]. Equal amounts of total protein were resolved by 10% SDS/PAGE in each experiment. Next, proteins were transferred to 0.45 µm polyvinylidene difluoride (PVDF) membrane (Millipore, ISEQ00010) and incubated with primary antibody against collagen I (ab254113, Abcam) and β-actin (66009-1-Ig, Proteintech, China) for 12–20 h at 4 °C. The membrane was incubated with HRP-linked secondary antibody followed by chemiluminescence detection (Millipore S.p.A., Italy).

### Real-time RT-PCR analysis of IL-6 mRNA expression

Total RNA was extracted from right lung tissues with a TRIzol kit (9108/9109, Takara, Japan) and quantified using Thermo BioMate3. Then, 1 μg of total RNA was transcribed into cDNAs using a PrimeScript™ RT reagent kit (RR047A, Takara, Japan) according to the manufacturer’s recommendations. Primers for IL-6 (forward: CTC CCA ACA GAC CTG TCT ATA C; reverse: CCA TTG CAC AAC TCT TTT CTC A) and GAPDH (forward: CAA CTA CAT GGT CTA CAT GTT C; reverse: CGC CAG TAG ACT CCA CGA C) were synthesized by Sangon Biotech (Shanghai, China). The RT-PCR assay was performed with TB Green™ Premix Ex Taq™II (RR820A, Takara, Japan) using an ABI 9600 real-time PCR detection system (Applied Biosystems). Three independent experiments were performed. β-Actin served as an endogenous control.

### Statistical analysis

All relevant data were analyzed using SPSS 2.0 software and reported as the means ± SD from three independently repeated experiments. N indicates the number of experiments repeated with the animals. Differences between mean values were analyzed using one-way ANOVA (Dunnett’s t-test) and two tailed Student’s t-test. *P* < 0.05 and *P* < 0.01 were considered statistically significant and extremely significant differences, respectively.

## Results

### Mice in the CS + LPS group presented a lower body weight than mice in the CS group

**Fig. 4:**
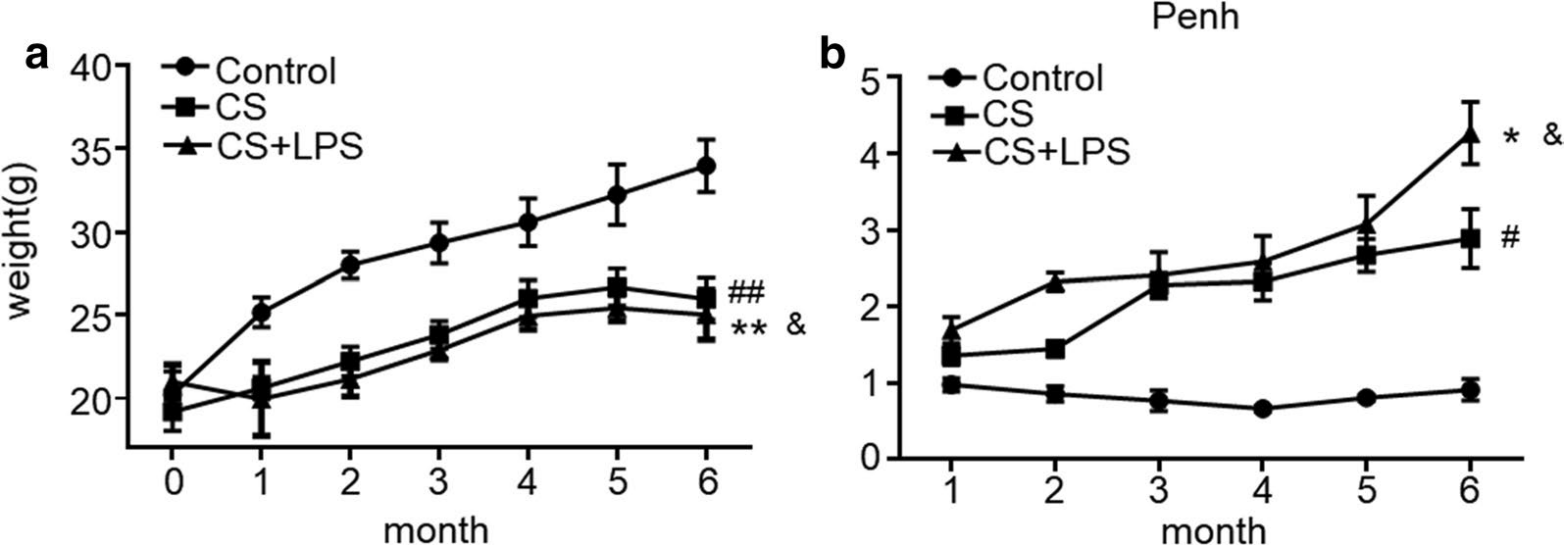
Measurements of the body weight and lung function each month. Six- to eight-week-old male C57BL/6J mice were exposed to CS for 6 months or CS for 6 months and an intranasal instillation of LPS on days 1 and 14. **a** Trends in the body weights of mice in the control group (n = 6), CS group (n = 6) and CS + LPS group (n = 6). **b** Lung function of mice in the control group (n = 6), CS group (n = 6) and CS + LPS group (n = 6). Penh, enhanced pause. Data are presented as the means ± SD, N = 6; ^#^*P* < 0.05; ^##^*P* < 0.01 for the comparison of the CS group with the control group; *P < 0.05; **P < 0.01; ***P < 0.001 for the comparison of the CS + LPS group with the control group; ^&^*P* < 0.05; ^&&^*P* < 0.01 for the comparison of the CS + LPS group with the CS group

During CS or CS + LPS exposure, the body weights of mice in both the CS group and CS + LPS group were lower than mice in the control group (*P* < 0.01), and the lowest body weights were recorded in the CS + LPS group (*P* < 0.05) (Fig. 4a). Interestingly, in the sixth month, the body weight of mice began to decrease in the CS and CS + LPS groups, but still increased in the control group.

### Mice in the CS + LPS group exhibited a more severe airflow limitation than mice in the CS group

Penh is an important and stable parameter that reflects airflow limitation. As shown in Fig. 4b, a progressive increase in Penh was observed in the CS and CS + LPS groups. Moreover, Penh increased earlier and more rapidly in mice in the CS + LPS group than in the CS group (*P* < 0.05). However, little change in Penh was observed in the control group during CS or CS + LPS exposure.

### Pulmonary inflammation increased more significantly in mice in the CS + LPS group than in mice in the CS group

**Fig. 5:**
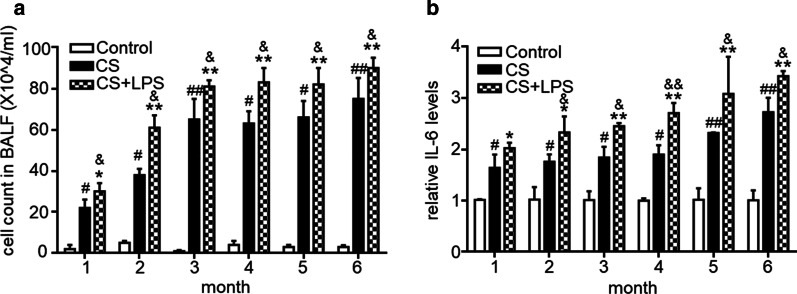
Number of total cells in the BALF and expression of the IL-6 mRNA in the mouse lung. Six- to eight-week-old male C57BL/6 J mice were exposed to CS for 6 months or CS for 6 months and an intranasal instillation of LPS on days 1 and 14. **a** Number of total cells in the BALF from mice sacrificed in the indicated month from the control group (n = 6), CS group (n = 6) and CS + LPS group (n = 6). **b** Relative IL-6 mRNA levels in the lungs of mice sacrificed in the indicated month from the control group (n = 6), CS group (n = 6) and CS + LPS group (n = 6). Data are presented as the means ± SD. ^#^*P* < 0.05; ^##^*P* < 0.01 for the comparison of the CS group with the control group; *P < 0.05; **P < 0.01; ***P < 0.001 for the comparison of the CS + LPS group with the control group; ^&^*P* < 0.05; ^&&^*P* < 0.01 for the comparison of the CS + LPS the group with the CS group

The cells in BALF from mice in the CS or CS + LPS group contained neutrophils, macrophages, lymphocytes, and a few bronchial epithelial cells. A noticeably greater number of total cells was observed in BALF from mice in the CS and CS + LPS groups than in the control group, and the highest numbers were detected in the CS + LPS group (*P* < 0.05, Fig. 5a). The number of total cells in BALF from mice in the CS and CS + LPS groups was markedly increased in the first 3 months and then maintained at a high level over the next 3 months. The IL-6 mRNA was expressed at higher levels in mouse lung tissues from both the CS and CS + LPS groups, and the highest levels were observed in the CS + LPS group, but a significant increasing trend was not observed with the exposure time (Fig. 5b).

### Greater amounts of collagen deposition were observed in the airways of the CS + LPS group than in the CS group

**Fig. 6:**
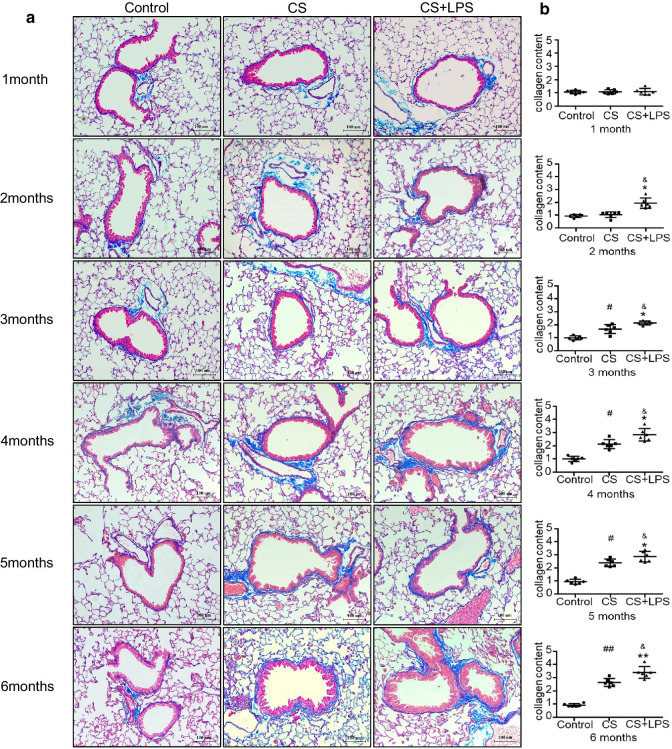
Collagen deposition around the airway, as assessed using Masson’s trichrome staining. Six- to eight-week-old male C57BL/6 J mice were exposed to CS for 6 months or CS for 6 months and an intranasal instillation of LPS on days 1 and 14. **a** Lung morphology was assessed by performing Masson’s trichrome staining of lung sections. Original magnification: × 200, bar = 100 μm; collagen: blue; nuclei: black; cytoplasm/epithelia: red. **b** Quantification of Masson’s trichrome staining for the collagen content in mice from the control group (n = 6), CS group (n = 6) and CS + LPS group (n = 6). Data are presented as the means ± SD, N = 6; ^#^*P* < 0.05; ^##^*P* < 0.01 for the comparison of the CS group with the control group; *P < 0.05; **P < 0.01; ***P < 0.001 for the comparison of the CS + LPS group with the control group; ^&^*P* < 0.05; ^&&^*P* < 0.01 for the comparison of the CS + LPS group with the CS group

In the present study, Masson’s trichrome staining of lungs from both the CS and CS + LPS groups showed collagen deposition, airway thickening and inflammatory cell infiltration compared to lungs from the control group. Moreover, compared with mice in the CS group, collagen deposition appeared earlier and greater amounts of collagen deposition were observed in the lung sections from mice in the CS + LPS group (Fig. 6a, b, *P* < 0.05). Collagen deposition is a recognized feature of airway remodeling and increased with the duration of exposure in both the CS and CS + LPS groups, but no obvious change was identified in the control group.

### Collagen deposition was greater around the pulmonary vessels of mice in the CS + LPS group than around those of mice in the CS group

**Fig. 7:**
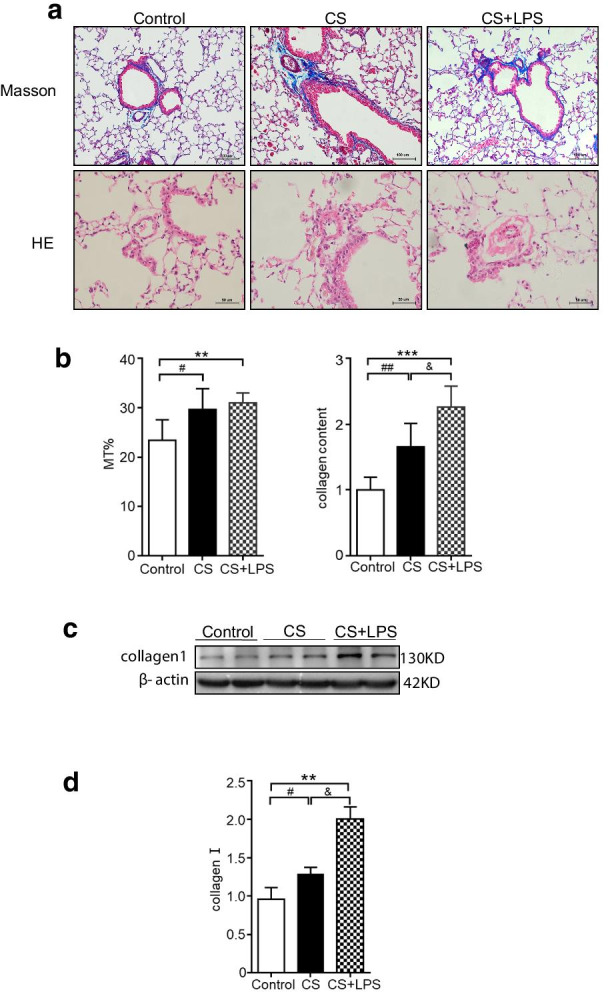
Assessment of pulmonary arteriole thickness and collagen content around arterioles by Masson’s trichrome staining, HE staining and Western blot. Six- to eight-week-old male C57BL/6 J mice were exposed to CS for 6 months or CS for 6 months and an intranasal instillation of LPS on days 1 and 14. **a** CS or CS + LPS exposure induced pulmonary arteriole thickening and deposition of collagen around arterioles. Masson staining of lung tissues from mice in the control group, CS group and CS + LPS group after 6 months of exposure, original magnification × 200. Bar = 100 μm. HE staining of lung tissues from mice in the control group, CS group and CS + LPS group after 6 months of exposure, original magnification × 400. Bar = 50 μm. **b** Quantification of collagen content around pulmonary arterioles and MT% of mice sacrificed after 6-month exposure from the control group (n = 6), CS group (n = 6) and CS + LPS group (n = 6). **c** Assessment of collagen I and β-actin by Western blot. The whole PVDF membrane was cropped at 70 kDa and full-length blots are presented in Additional file 1: Figure S1. **d** Determination of protein levels of collagen I. Data are presented as the means ± SD, N = 6; ^#^*P* < 0.05; ^##^*P* < 0.01 for the comparison of the CS group with the control group; *P < 0.05; ** *P* < 0.01; ***P < 0.001 for the comparison of the CS + LPS group with the control group. ^&^*P* < 0.05; ^&&^*P* < 0.01 for the comparison of the CS + LPS group with the CS group

No significant difference in pulmonary vessels among the groups appeared until the mice were exposed to cigarette smoke for 6 months or more. Shown in Fig. 7a–d, the Masson staining revealed that after 6-month exposure, the collagen content around the arterioles in the CS + LPS group was greater than that around the arterioles in the CS group (*P* < 0.05). In consistent with Masson staining, collagen Iof mouse lung in CS and CS + LPS groups increase could be assessed by western blot, in addition, collagen in CS + LPS group increased more significantly than CS group, full-length blots/gels are presented in Additional file 1: Figure S1.

HE staining showed that the pulmonary arteriole thickness of mice in both the CS and CS + LPS groups was thicker than that in mice in the control group (*P* < 0.05), but arteriole thickness between the two model groups was not significantly different.

### The airspace enlargement of mice in the CS + LPS group was similar to mice in the CS group

**Fig. 8:**
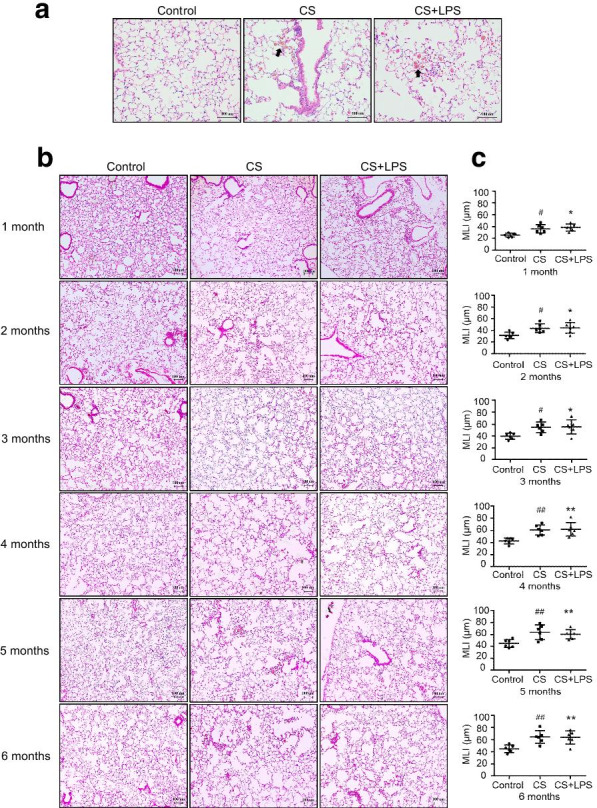
Airspace enlargement assessed using H&E staining. Six- to eight-week-old male C57BL/6 J mice were exposed to CS for 6 months or CS for 6 months and an intranasal instillation of LPS on days 1 and 14. **a** CS or CS + LPS exposure induced inflammation in the lung. H&E staining of lung tissues from mice in the control group, CS group and CS + LPS group after 6 months of exposure, original magnification × 200. Bar = 100 μm. The arrows indicate inflammatory cells. **b** H&E staining of lung tissues from mice sacrificed in the indicated month from the control group (n = 6), CS group (n = 6) and CS + LPS group (n = 6), original magnification × 100. Bar = 100 μm. **c** Quantification of the MLI of mice sacrificed in the indicated month from the control group (n = 6), CS group (n = 6) and CS + LPS group (n = 6). Data are presented as the means ± SD, N = 6; ^#^*P* < 0.05; ^##^*P* < 0.01 for the comparison of the CS group with the control group; *P < 0.05; **P < 0.01; ***P < 0.001 for the comparison of the CS + LPS group with the control group

As shown in Fig. 8a, lung sections from mice in the CS and CS + LPS groups showed inflammatory cell interstitial infiltration and alveolar fusion. As shown in Fig. 8b, c, compared with the control group, the MLI increased after 1 month of exposure and the most marked change was noted after 4 months of exposure in the CS and CS + LPS groups (*P* < 0.05 or *P* < 0.01), but the difference in MLI between the two groups was not significant. In lung sections from mice in the control group, the MLI also increased as the mice aged, although mice in the control group were not exposed to CS or LPS.

## Discussion

Chronic inflammation, emphysema and airway remodeling are the main characteristics of COPD, which induce airflow limitation and cause patients with COPD to suffer from various symptoms [18]. The establishment of an appropriate mouse model is necessary to explore the mechanism of COPD. Because CS and LPS exposure are both recognized as key risk factors for COPD, FengZhou studied on the final pathological changes of COPD models induced by CS and LPS, Jiaze Shu compared whole-body CS exposure with nose-only CS exposure combined with LPS inhalation [13, 14]. In this study, we first studied the dynamic evolution of emphysema and airway remodeling in two mouse models of COPD induced by CS and CS + LPS to explore the law of COPD modeling [14, 19]. Based on the results of our study, both CS and CS + LPS induce COPD in a mouse model, and lung function, airway inflammation and collagen deposition in mice exposed to CS or CS + LPS are positively correlated with exposure time. However, the lung function in mice in the CS + LPS group decreased more significantly, pulmonary inflammation was more severe, and the collagen content increased earlier and to a greater extent than in mice in the CS group. Nevertheless, emphysema can be caused by relatively short exposure time, while a longer exposure time will not cause much progression. Moreover, few differences in emphysema were observed between mice in the CS and CS + LPS groups.

CS contains more than 4000 different chemicals, which are deposited in the small airways to cause a chronic inflammatory response and generate many oxidants, inducing irreversible lung tissue damage [20]. LPS, a component of gram-negative bacteria, is a strong pro-inflammatory endotoxin [21, 22]. Therefore, the CS + LPS method simulates an infection-induced acute exacerbation of COPD to some extent. In the present study, intranasal instillations of LPS were administered in the first 2 weeks, but the inflammation induced by LPS appeared to be maintained during CS exposure. The total cell numbers in the BALF and IL-6 levels in lungs from CS + LPS-exposed mice were higher than in CS-exposed mice. Because collagen deposition is one of the most characteristic pathological changes of airway remodeling [23], we estimated airway remodeling by measuring collagen deposition around the airway. CS and LPS both induce airway remodeling through inflammatory and non-inflammatory mechanisms. CS and LPS may induce airway remodeling independent of inflammation via common pathways, and CS combined with LPS does not exert an additive effect compared with CS exposure alone in vitro [24]. In the present study, collagen deposition around the airways of CS + LPS-exposed mice not only increased earlier but also was always present at higher levels than in CS-exposed mice, suggesting that CS + LPS may induce more severe airway remodeling by promoting inflammation. Although the widely accepted method of 6 months of CS exposure causes obvious collagen deposition, collagen levels increased after 2 months of CS + LPS exposure or 3 months of CS exposure in the present study. Emphysema results from alveolar apoptosis and senescence induced by oxidative stress and chronic inflammation [25, 26]. CS exposure for longer than 3 months induces emphysema in mice [27]. Our results were consistent with these findings. Compared with mice in the control group, the MLI started to increase after 1 month of exposure, and significant emphysema was observed in CS- and CS + LPS-exposed mice after 3 months. Interestingly, we also observed significant MLI progression in the first 4 months of exposure, but no obvious changes in the following months in either the CS group or the CS + LPS group. Moreover, the emphysema occurring in CS + LPS-exposed mice was not substantially different emphysema in CS-exposed mice.

In the present study, a non-invasive lung function test was used to assess the airflow limitation of mice. As an irreplaceable parameter for evaluation of the COPD mouse model, lung function can be tested by invasive techniques and noninvasive techniques. The invasive techniques are sensitive, specific and complicated to perform. Moreover, the same mouse cannot be repeatedly tested with invasive techniques. A slight airflow limitation of CS- or CS + LPS-exposed mice appeared after 1 month of exposure. The decrease in lung function was also consistent with the increased MLI and collagen deposition around the airway. Mice in the CS + LPS group with greater collagen deposition and similar MLI exhibited a more severe airflow limitation than mice in the CS group, suggesting that airflow limitation may mainly be induced by airway remodeling.

In addition, the weight measurement was similar to the results of a study by He et al. [16]. The weights of mice in the CS and CS + LPS groups were significantly lower than mice in the control group, consistent with the body weight loss of patients with COPD. However, the reduction appeared after only 1 month of exposure, and the mice in the CS + LPS group even exhibited weight loss. We hypothesize that the weight loss observed at an early stage does not result from COPD but is instead due to an inadaptability to exposure or addiction to nicotine. Moreover, the MLI of H&E staining in mice from the control group increased with age, suggesting that an increased MLI is induced by the development and aging of the lung.

In conclusion, we systematically and dynamically evaluated and compared the lung function and lung pathology of CS- and CS + LPS-exposed mice. A mouse model of COPD was established using both methods. The two methods induced emphysema in mice at an early stage, but CS + LPS exposure increased collagen deposition around the airway at an earlier time point than CS exposure. In summary, CS + LPS exposure is a more suitable method for analyzing the mechanism of airway remodeling, and exposure to CS alone is more suitable for emphysema research due to its simple operation.

## Supplementary Information

**Additional file 1: Figure S1.** The original image of the western blot. **(A)** The original image of collagen I. **(B)** The original image of β-actin. **(C)** The full-length blots with 6 sample lanes and 2 markers (Thermo 26616, USA).

## Data Availability

All data analyzed during this study are included in this article and its figures. All processed data and models used in the study are available from the corresponding author upon request.

## References

[1] Riley CM, Sciurba FC (2019). Diagnosis and outpatient management of chronic obstructive pulmonary disease: a review. JAMA.

[2] De Smet EG, Van Eeckhoutte HP, Avila CF, Blomme E, Verhamme FM, Provoost S, Bracke KR (2020). The role of miR-155 in cigarette smoke-induced pulmonary inflammation and COPD. Mucosal Immunol..

[3] Singh D, Agusti A, Anzueto A, Barnes PJ, Bourbeau J, Celli BR, Vogelmeier C (2019). Global strategy for the diagnosis, management, and prevention of chronic obstructive lung disease: the GOLD science committee report 2019. Eur Respir J.

[4] Holgate S, Agusti A, Strieter RM, Anderson GP, Fogel R, Bel E, Reiss TF (2015). Drug development for airway diseases: looking forward. Nat Rev Drug Discov.

[5] Fricker M, Deane A, Hansbro PM (2014). Animal models of chronic obstructive pulmonary disease. Expert Opin Drug Discov.

[6] Leberl M, Kratzer A, Taraseviciene-Stewart L (2013). Tobacco smoke induced COPD/emphysema in the animal model-are we all on the same page?. Front Physiol.

[7] Hikichi M, Mizumura K, Maruoka S, Gon Y (2019). Pathogenesis of chronic obstructive pulmonary disease (COPD) induced by cigarette smoke. J Thorac Dis.

[8] Aghapour M, Raee P, Moghaddam SJ, Hiemstra PS, Heijink IH (2018). Airway epithelial barrier dysfunction in chronic obstructive pulmonary disease: role of cigarette smoke exposure. Am J Respir Cell Mol Biol.

[9] Yoshida M, Minagawa S, Araya J, Sakamoto T, Hara H, Tsubouchi K, Kuwano K (2019). Involvement of cigarette smoke-induced epithelial cell ferroptosis in COPD pathogenesis. Nat Commun.

[10] Tam A, Churg A, Wright JL, Zhou S, Kirby M, Coxson HO, Sin DD (2016). Sex differences in airway remodeling in a mouse model of chronic obstructive pulmonary disease. Am J Respir Crit Care Med.

[11] Han W, Dong Z, Dimitropoulou C, Su Y (2011). Hydrogen sulfide ameliorates tobacco smoke-induced oxidative stress and emphysema in mice. Antioxid Redox Signal.

[12] Xu H, Ling M, Xue J, Dai X, Sun Q, Chen C, Liu Q (2018). Exosomal microRNA-21 derived from bronchial epithelial cells is involved in aberrant epithelium-fibroblast cross-talk in COPD induced by cigarette smoking. Theranostics.

[13] Zhou F, Li DF, Yuan L, Fu HY, Ma P, Zhang KD, Lu WJ (2019). A comparative study of two chronic obstructive pulmonary disease mouse models established by different methods. Zhonghua Jie He He Hu Xi Za Zhi.

[14] Shu J, Li D, Ouyang H, Huang J, Long Z, Liang Z, Lu W (2017). Comparison and evaluation of two different methods to establish the cigarette smoke exposure mouse model of COPD. Sci Rep.

[15] Di T, Yang Y, Fu C, Zhang Z, Qin C, Sai X, Bian T (2021). Let-7 mediated airway remodelling in chronic obstructive pulmonary disease via the regulation of IL-6. Eur J Clin Invest..

[16] He S, Li L, Sun S, Zeng Z, Lu J, Xie L (2018). A novel murine chronic obstructive pulmonary disease model and the pathogenic role of MicroRNA-21. Front Physiol.

[17] Zhang M, Feng Z, Huang R, Sun C, Xu Z (2018). Characteristics of pulmonary vascular remodeling in a novel model of shunt-associated pulmonary arterial hypertension. Med Sci Monit.

[18] Rossi A, Butorac-Petanjek B, Chilosi M, Cosio BG, Flezar M, Koulouris N, Miravitlles M (2017). Chronic obstructive pulmonary disease with mild airflow limitation: current knowledge and proposal for future research—a consensus document from six scientific societies. Int J Chron Obstruct Pulmon Dis.

[19] Kimura A, Yamauchi Y, Hodono S, Stewart NJ, Hosokawa O, Hagiwara Y, Fujiwara H (2017). Treatment response of ethyl pyruvate in a mouse model of chronic obstructive pulmonary disease studied by hyperpolarized (129) Xe MRI. Magn Reson Med.

[20] Angelis N, Porpodis K, Zarogoulidis P, Spyratos D, Kioumis I, Papaiwannou A, Zarogoulidis K (2014). Airway inflammation in chronic obstructive pulmonary disease. J Thorac Dis.

[21] Brass DM, Hollingsworth JW, Cinque M, Li Z, Potts E, Toloza E, Schwartz DA (2008). Chronic LPS inhalation causes emphysema-like changes in mouse lung that are associated with apoptosis. Am J Respir Cell Mol Biol.

[22] Li Z, Wang J, Wang Y, Jiang H, Xu X, Zhang C, Lu W (2014). Bone morphogenetic protein 4 inhibits liposaccharide-induced inflammation in the airway. Eur J Immunol.

[23] Hirota N, Martin JG (2013). Mechanisms of airway remodeling. Chest.

[24] Pera T, Gosens R, Lesterhuis AH, Sami R, van der Toorn M, Zaagsma J, Meurs H (2010). Cigarette smoke and lipopolysaccharide induce a proliferative airway smooth muscle phenotype. Respir Res.

[25] Bodas M, Min T, Vij N (2011). Critical role of CFTR-dependent lipid rafts in cigarette smoke-induced lung epithelial injury. Am J Physiol Lung Cell Mol Physiol.

[26] Bodas M, Min T, Vij N (2015). Lactosylceramide-accumulation in lipid-rafts mediate aberrant-autophagy, inflammation and apoptosis in cigarette smoke induced emphysema. Apoptosis.

[27] Mizutani N, Fuchikami J, Takahashi M, Nabe T, Yoshino S, Kohno S (2009). Pulmonary emphysema induced by cigarette smoke solution and lipopolysaccharide in guinea pigs. Biol Pharm Bull.

