# Supplementary figures and images for: Dynamic evolution of emphysema and airway remodeling in two mouse models of COPD
Source: BMC Pulm Med. 2021 Apr 26;21:134. doi: 10.1186/s12890-021-01456-z (PMC8073949; doi:10.1186/s12890-021-01456-z)

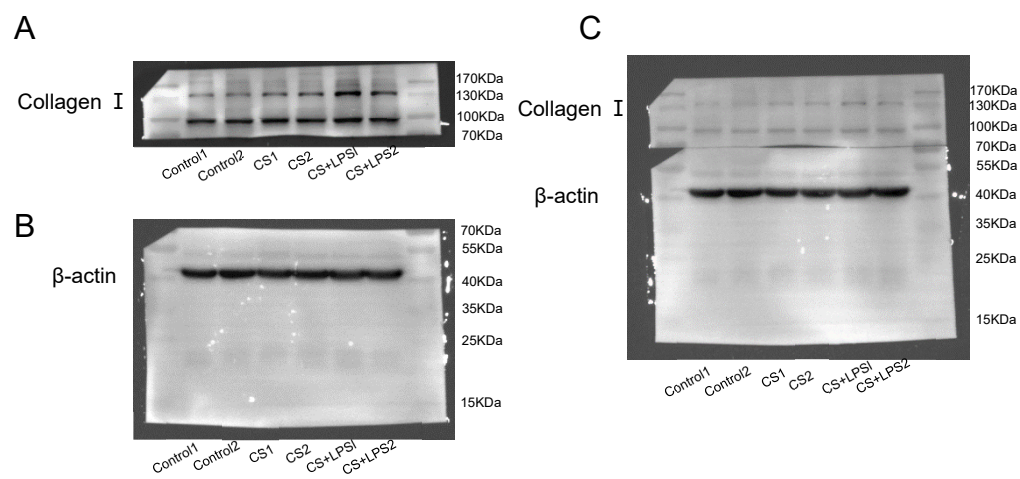

Supplementary Figure 1

Supplement: Supplementary file 1 — Additional file 1: Figure S1. The original image of the western blot. (A) The original image of collagen I. (B) The original image of β-actin. (C) The full-length blots with 6 sample lanes and 2 markers (Thermo 26616, USA). [file 12890_2021_1456_MOESM1_ESM.pdf]
